# Supplementary material for: Perceptual bias is reduced with longer reaction times during visual discrimination
Source: Commun Biol. 2020 Feb 10;3:59. doi: 10.1038/s42003-020-0786-7 (PMC7010686; doi:10.1038/s42003-020-0786-7)
Supplement: Supplementary file 1 — Supplementary Information [file 42003_2020_786_MOESM1_ESM.pdf]

Supplementary Figures

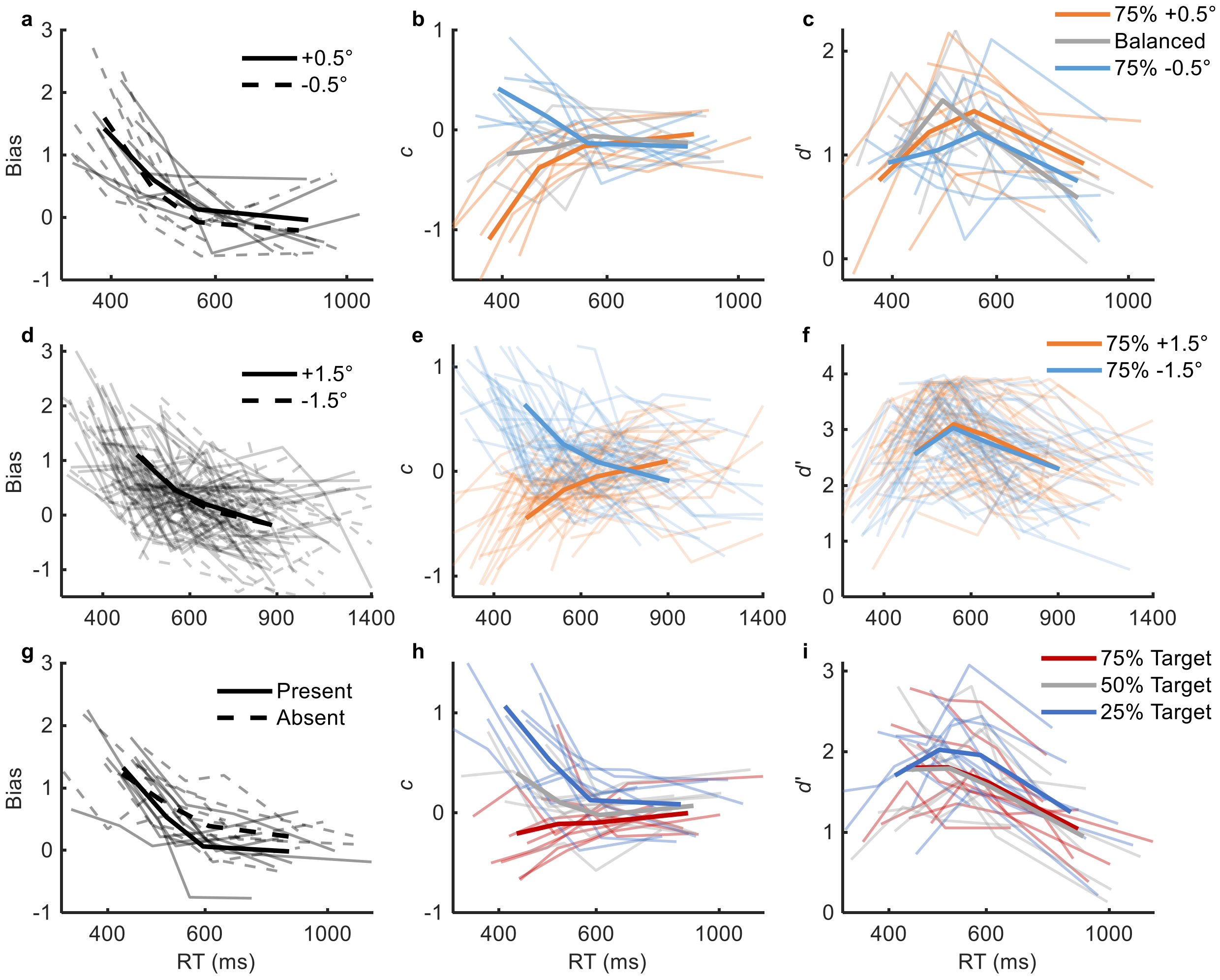

Supplementary Figure 1 – Individual data in the prior-dependent experiments. Shown for the discrimination experiment, for each observer (transparent lines) and averaged across observers (heavy opaque lines), are (**a**) bias for the different stimuli (line styles), (**b**) internal criterion for the different priors (colors), and (**c**) sensitivity (*d*'), as a function of RT, in four equal-quantity bins. (**d-f**) Same for the "Discrimination MTurk" experiment. (**g-i**) Same for the detection experiment. The average data are reproduced from Fig. 3.

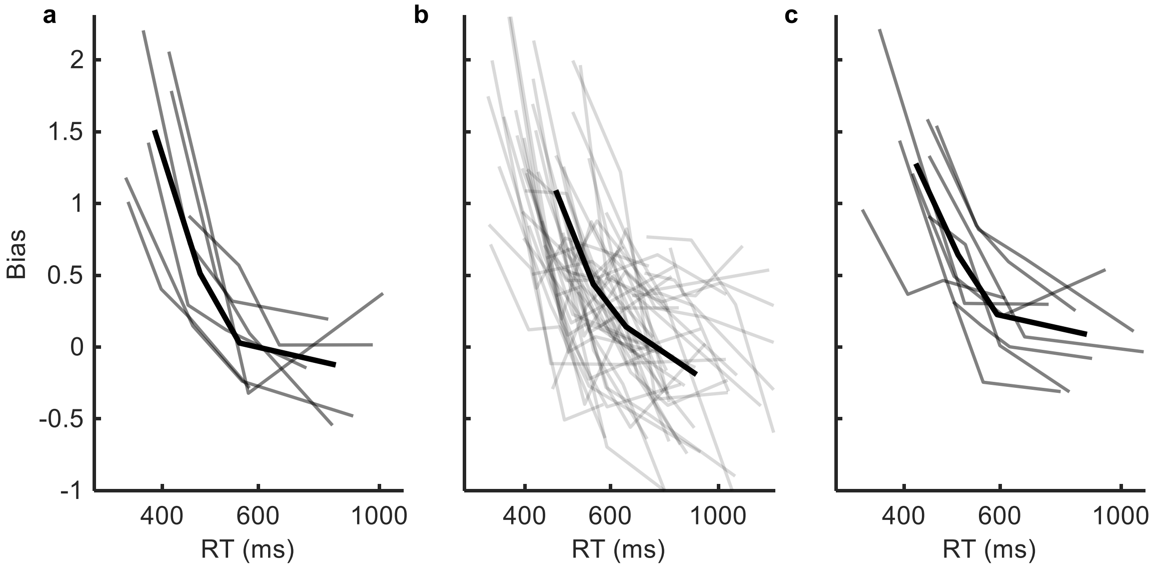

Supplementary Figure 2 – Individual bias data in the prior-dependent experiments. Shown for each observer (gray) and averaged across observers (black) is bias as a function of RT, averaged over the two stimuli alternatives (Supplementary Fig. 1adg), in four equal-quantity bins, for the (**a**) "Discrimination", (**b**) "Discrimination MTurk", and (**c**) "Detection" experiments. Results showed negative correlation between bias and RT bin index. Note that binning is done separately for each observer, followed by averaging of bias and RT in each bin. The average data are reproduced from Fig. 7a, but plotted as a function of physical, rather than relative, time.

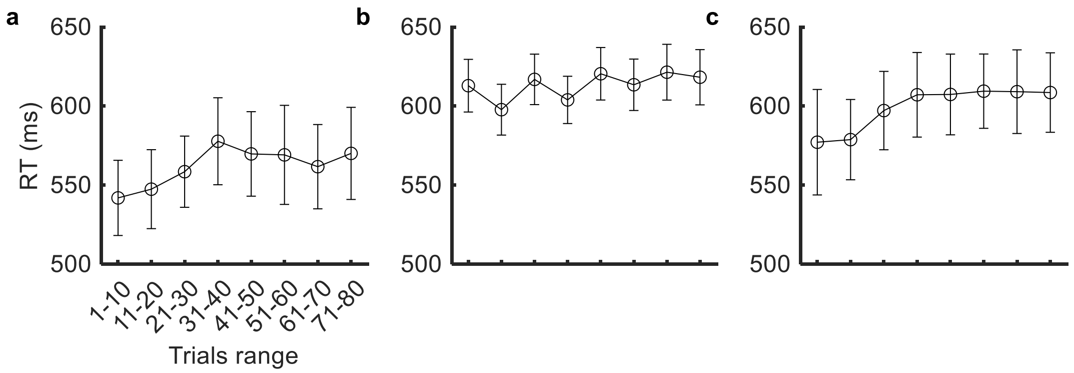

Supplementary Figure 3 – Learning control for the prior-dependent experiments. Shown is RT as a function of trial index within block (80 trials), averaged across block repetitions and then across observers, for the (**a**) "Discrimination", (**b**) "Discrimination MTurk", and (**c**) "Detection" experiments. It can be seen that RT is non-decreasing, possibly increasing, within block. Therefore, based on a trial's position within a block, and because prior is gradually learned, we would have expected a positive correlation between RT and bias, unlike observed behaviorally (Figs. 1-3, Supplementary Figs. 1 and 2). This finding controls for the potential confound of gradual learning of the prior. Note that the first ten trials were excluded from all other analyses (see Methods). Error bars are ±1*SEM*.

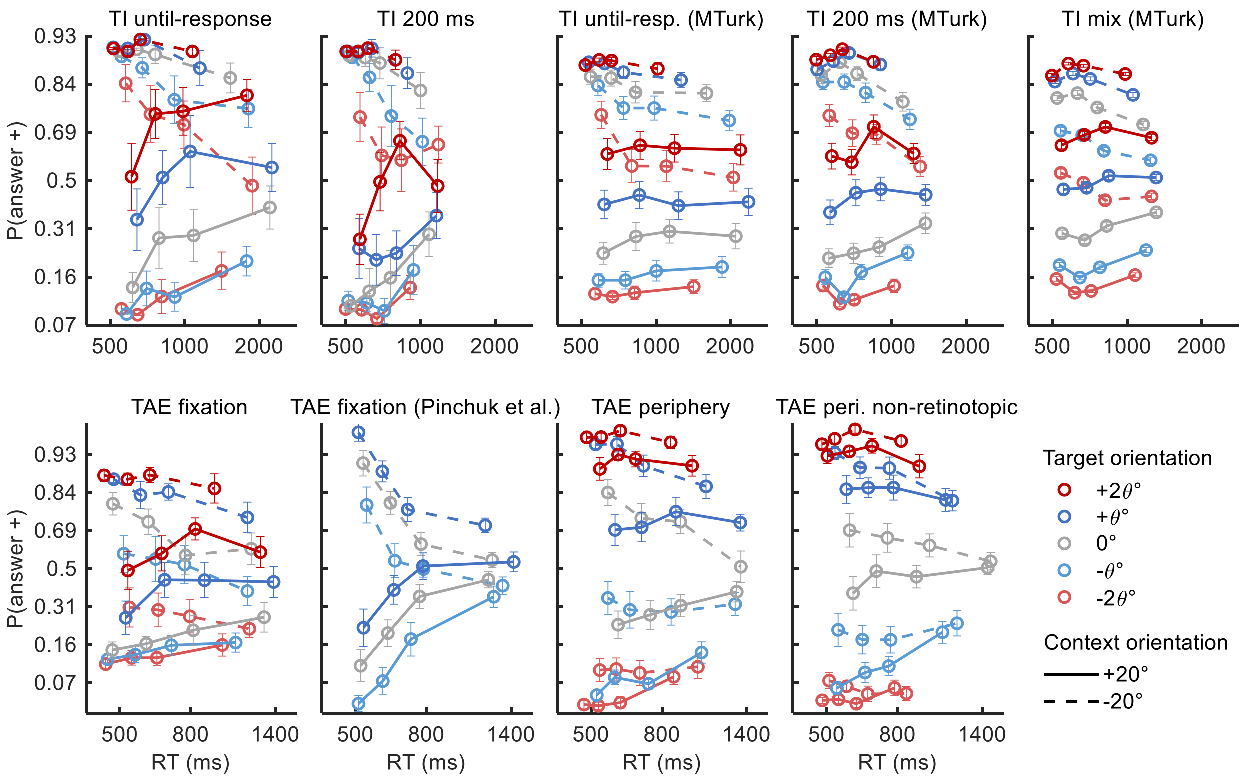

Supplementary Figure 4 – Interaction of bias and time for TI and TAE. Shown is the response probability as a function of reaction time, under different context orientations (line styles: -20° is dashed, +20° is solid), and different physical target orientations (colors: gray, blue, and red indicate 0°, ±θ°, and ±2θ° target orientations; θ = 1° for TI and TAE fixation, and θ = 2° for TAE periphery; data from the TI MTurk experiments with *3*° target orientation steps is not shown). Bias is the vertical distance between data points of the same color with different line styles. The presence of intersections between lines corresponding to different target (color) and context (line style) orientations suggests that the context-dependent bias was time-dependent (e.g., an intersection between the dashed gray line and solid blue line in the TAE periphery condition can be taken as evidence that the 0° target with a clockwise context-induced bias is not interchangeable with the 2° target with a counter-clockwise bias). Error bars are ±1*SEM*.

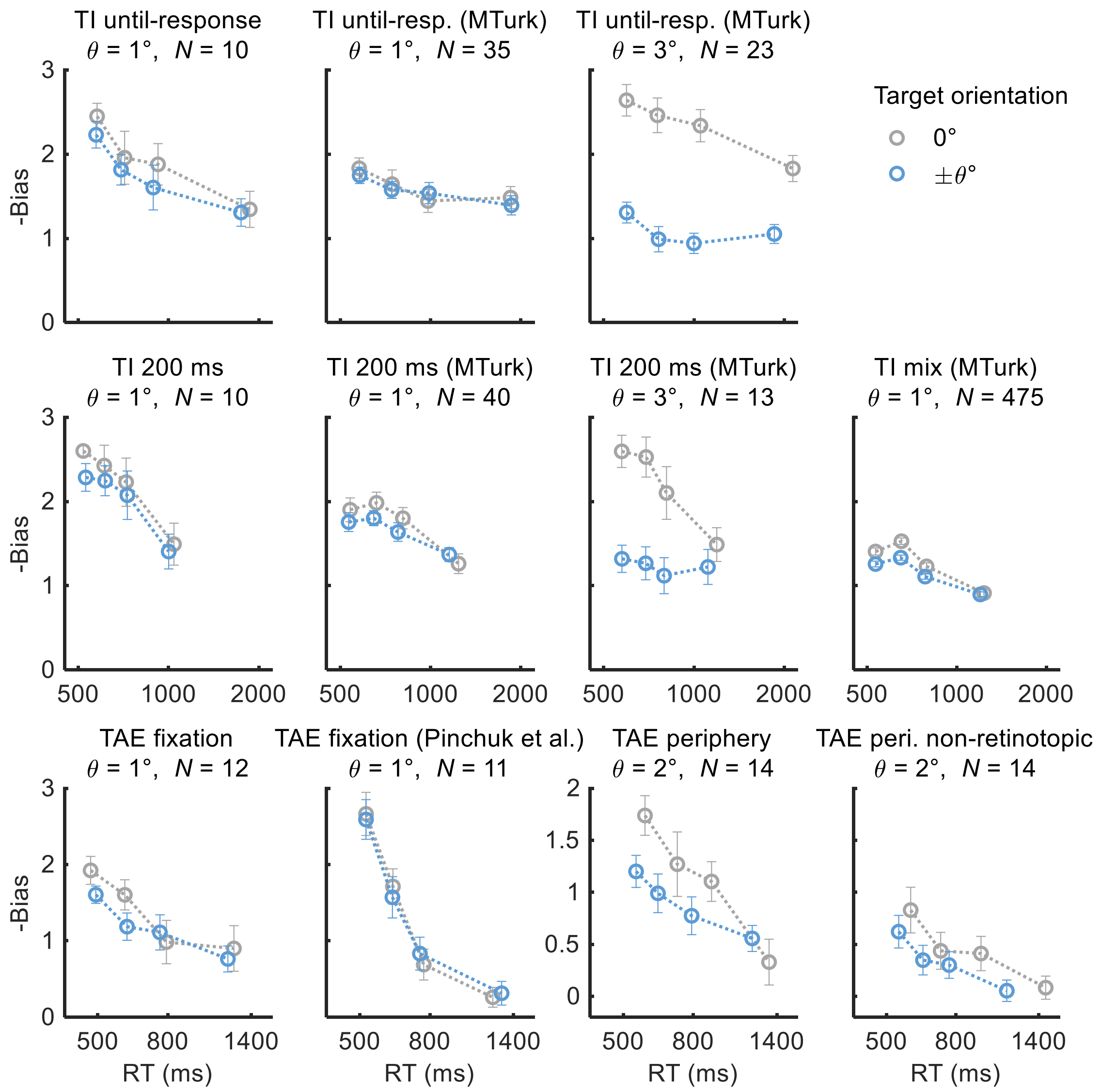

Supplementary Figure 5 –Bias in single, physical orientations for the context-dependent experiments. Shown is the average across observers of bias (equation (1)) under the different context conditions. Target orientations were denoted by color, where gray indicates 0°, and blue indicates an average of the +θ° and the -θ° measurements where θ is the step size between adjacent target orientations (θ = 1°, 2°, or 3°, see the Methods; note that two MTurk TI experiments appear twice, having both θ = 1° and θ = 3° versions). Results show reduction in bias across experiments and physical target orientations. Note that unlike Fig. 4, here bias is calculated for single, fixed target orientations (not shifted to near the PV, and no pooling of a pair of target orientations), leading to reduced measurable effects when measurements saturate (see the Methods). Error bars are ±1*SEM*.

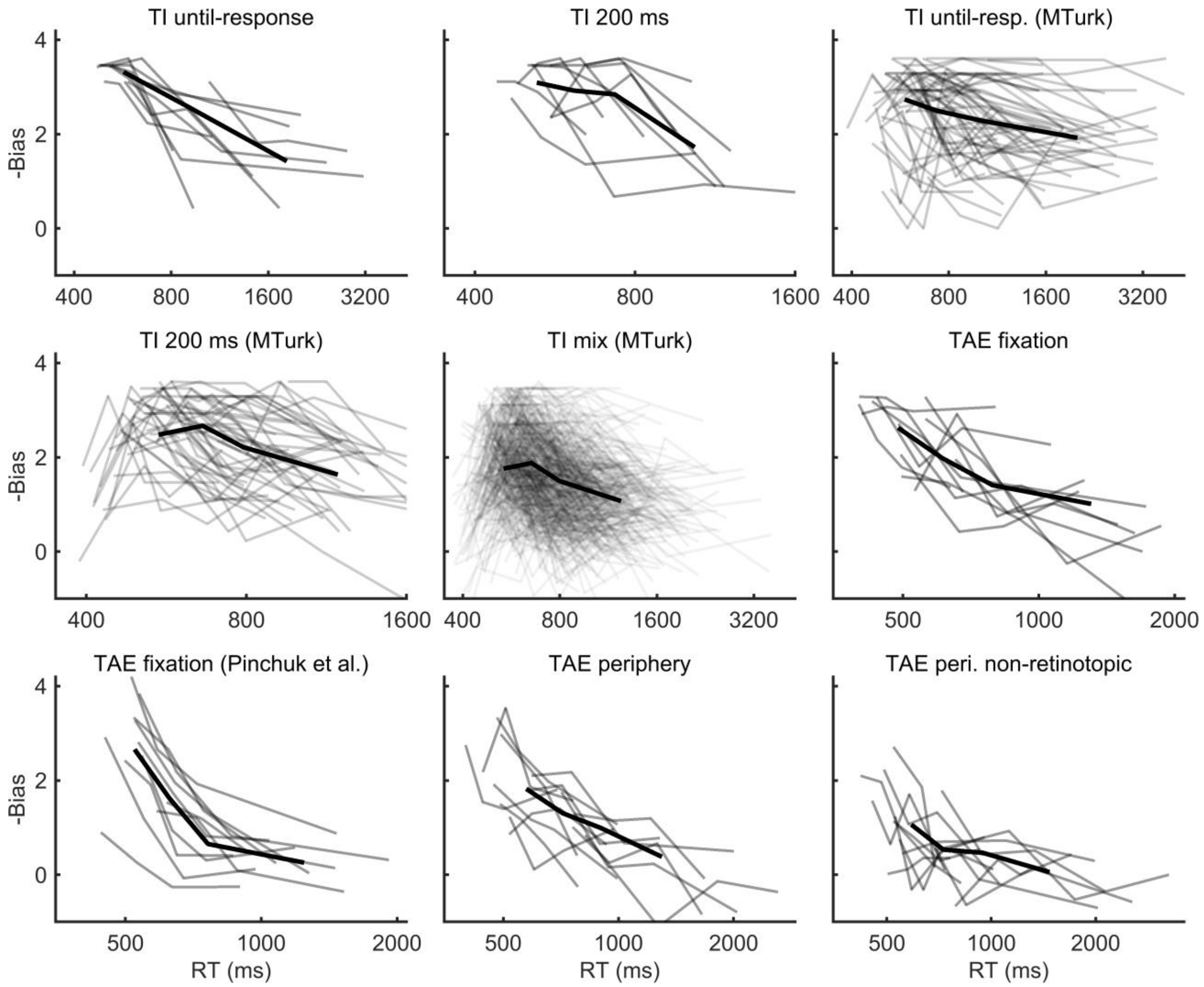

Supplementary Figure 6 – Individual bias data in the context-dependent experiments. Shown for each observer (gray) and averaged across observers (black) is bias as a function of RT, in four approximately equal quantity bins. Results showed negative correlation between bias and RT bin index. Note that binning is done separately for each observer, and then bias and RT are averaged for each of the four bins. The average data are reproduced from Figs. 4 and 8.

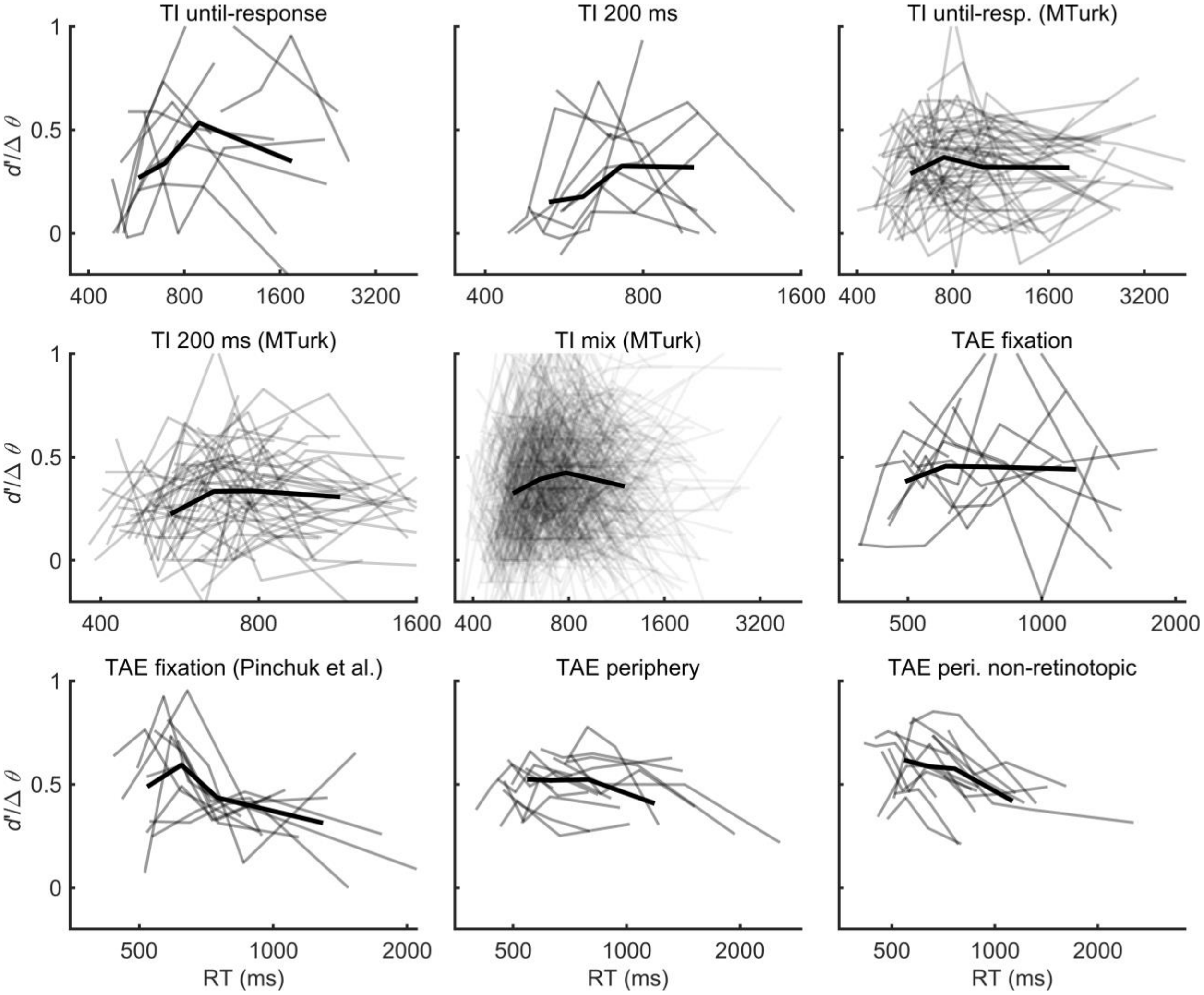

Supplementary Figure 7 – Individual sensitivity data in the context-dependent experiments. Shown for each observer (gray) and averaged across observers (black) is *d'* divided by the orientation difference ($\Delta\theta = 2\theta$) and averaged across the two contexts, as a function of RT, in four approximately equal quantity bins. The average data are reproduced from Fig. 5.

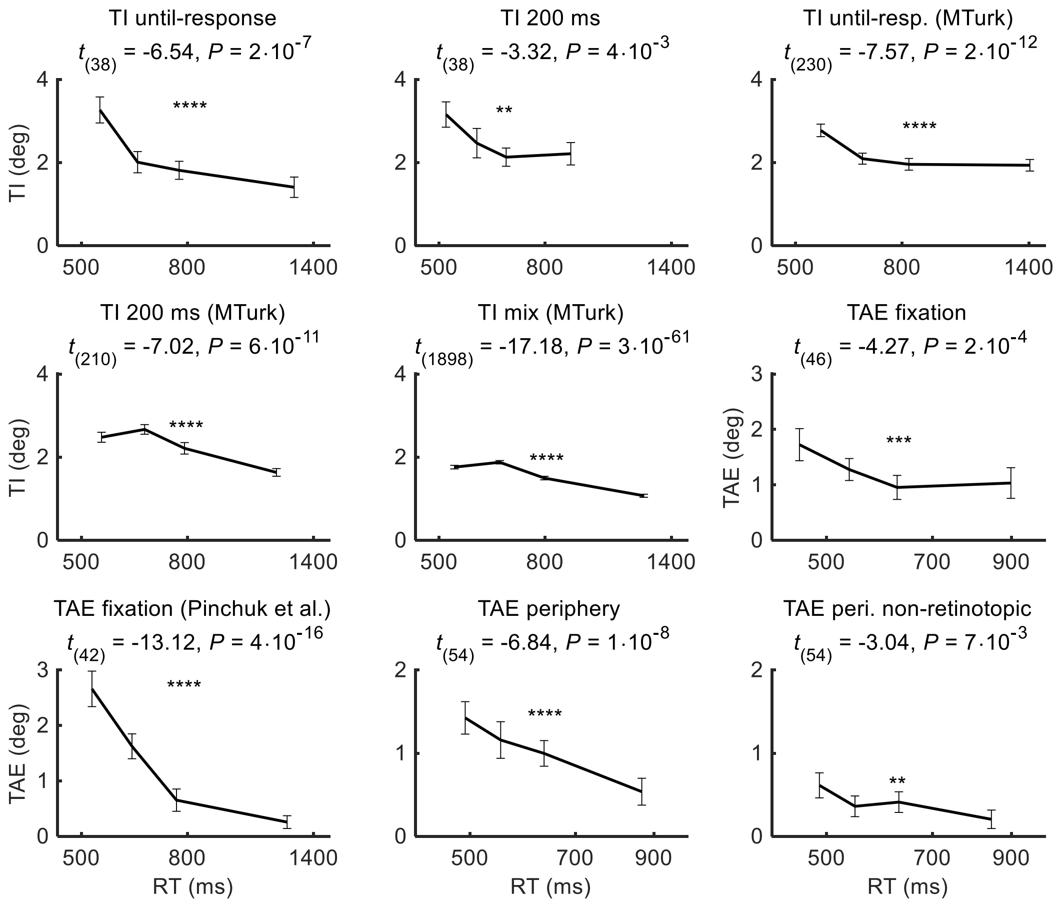

Supplementary Figure 8 – Magnitude of TAE and TI in degrees. Shown is bias measured as half the shift in the perceived vertical target orientation due to the context orientation (adaptor or surround, for TAE or TI, respectively, averaged across observers), as a function of the mean RT (first averaged separately for each target orientation, then across orientations, then across observers). Measurements were obtained by fitting a cumulative normal distribution to the psychometric function of percent clockwise reports as a function of target orientation (see Methods). Note that this method of analysis is less accurate than bias (equation (1)) for analyzing an interaction with RT, because different target orientations reflect different difficulty levels, hence different RTs. Still, to ensure a balanced number of trials per target orientation in an RT bin, the binning here was performed separately for each combination of context orientation and target orientation. Error bars are ±1*SEM*, and asterisks indicate the significance level of the change in bias magnitude in different RT bins obtained using a linear mixed-effects regression as in the main text (Bonferroni corrected for two multiple comparisons; ** $p < 0.01$, *** $p < 0.001$, **** $p < 0.0001$).

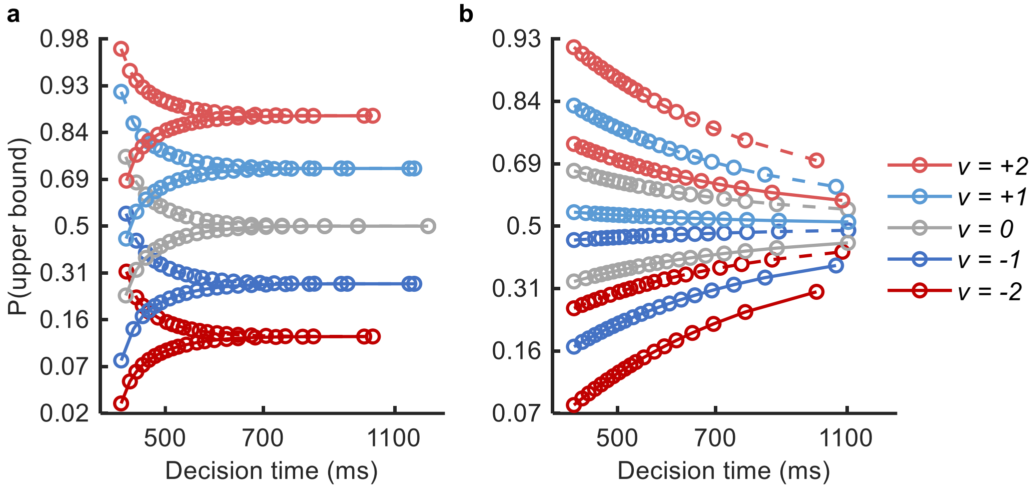

Supplementary Figure 9 – Against an account of reduction in bias from inter-trial variability. Shown is the percent of trials that reach the upper bound in a given decision-time bin, when the influence of context or prior (different line styles) is modeled using the drift diffusion model (DDM) as (**a**) a change in the starting point ($z = 0.5 \pm 0.05$, where the bounds are at 0 and 1), or (**b**) a change in the drift rate ($v = v \pm 0.8$) with added inter-trial variability (standard deviation of drift rate, *sv*, taking a value of 2)[1]. The influence of different target orientations was modeled as different drift rates (baseline values of *v*, as shown in the legend). Both models show a reduction in bias as a function of time. Importantly, in the drift rate version (panel **b**), the modeled context influence is obviously interchangeable with a change in the drift rate, hence there are no line intersections. This illustrates the idea that when the influence of context or prior is not interchangeable with a change in evidence (e.g., change in orientation of target), then the contextual influence is time-dependent (e.g., change in starting point). Note that the exact modeling details for the contextual influence are described in the main text; this figure illustrates the above idea.

Supplementary Tables

Supplementary Table 1 – Statistics for a change in bias with RT.

Experiment		N	Linear mixed-effects regression (slope term for bias)				Paired difference of first and last RT bins			
			N effect	*df*	*t*	*P*	*M*	*SD*	Cohen's *d*	Hedges' *g*
Prior-dependent	Discrimination	7	7	26	-8.68	$7 \cdot 10^{-9}$	1.64	0.46	3.53	3.14
	Discrimination MTurk	50	48	198	-14.60	$6 \cdot 10^{-33}$	1.28	0.80	1.60	1.57
	Detection	9	9	34	-8.89	$4 \cdot 10^{-10}$	1.19	0.52	2.27	2.07
Context-dependent	TI until-response	10	10	38	7.65	$7 \cdot 10^{-9}$	-1.89	0.68	2.78	2.57
	TI 200 ms	10	9	38	4.23	0.0003	-1.37	1.11	1.24	1.14
	TI MTurk until-response	58	45	230	7.51	$3 \cdot 10^{-12}$	-0.82	0.87	0.93	0.92
	TI MTurk 200 ms	53	45	210	7.02	$6 \cdot 10^{-11}$	-0.85	1.01	0.84	0.83
	TI MTurk mix	475	386	1898	17.18	$3 \cdot 10^{-61}$	-0.69	1.04	0.66	0.66
	TAE fixation	12	11	46	6.54	$9 \cdot 10^{-8}$	-1.61	0.79	2.03	1.90
	TAE fixation Pinchuk et al.[2]	11	11	42	13.12	$4 \cdot 10^{-16}$	-2.40	0.85	2.83	2.63
	TAE periphery	14	14	54	7.35	$2 \cdot 10^{-9}$	-1.44	0.78	1.85	1.75
	TAE periphery non-retinotopic	14	11	54	4.35	$1 \cdot 10^{-4}$	-1.01	1.13	0.90	0.85

The linear mixed-effects regression was as described in the Methods section (with RT bin index as the regressor). Reported *P* values are Bonferroni corrected for two multiple comparisons. The "*N* effect" shows the number of observers with a slope sign indicating a reduction in bias with time. Note that bias is positive for the prior-dependent experiments, and negative for the context-dependent experiments.

Supplementary Table 2 – Statistics for the RT-independent bias.

Experiment		*N*	*N* effect	*M*	*SD*	Cohen's *d*	*t*-test vs. 0		
							df	*t*	*P*
Prior-dependent	Discrimination	7	7	0.43	0.23	1.87	6	4.95	$3 \cdot 10^{-3}$
	Discrimination MTurk	50	36	0.25	0.36	0.70	49	4.94	$1 \cdot 10^{-5}$
	Detection	9	9	0.49	0.26	1.85	8	5.55	$5 \cdot 10^{-4}$
Context-dependent	TI until-response	10	10	-2.68	0.65	4.11	9	-12.99	$4 \cdot 10^{-7}$
	TI 200 ms	10	10	-2.75	0.80	3.44	9	-10.87	$2 \cdot 10^{-6}$
	TI MTurk until-response	58	58	-2.53	0.93	2.73	57	-20.75	$3 \cdot 10^{-28}$
	TI MTurk 200 ms	53	53	-2.31	0.86	2.69	52	-19.61	$1 \cdot 10^{-25}$
	TI MTurk mix	475	474	-1.56	0.79	1.97	474	-42.99	$1 \cdot 10^{-165}$
	TAE fixation	12	12	-1.83	1.05	1.75	11	-6.07	$8 \cdot 10^{-5}$
	TAE fixation Pinchuk et al.[2]	11	11	-1.11	0.52	2.13	10	-7.05	$3 \cdot 10^{-5}$
	TAE periphery	14	13	-1.02	0.73	1.40	13	-5.24	$2 \cdot 10^{-4}$
	TAE periphery non-retinotopic	14	12	-0.45	0.40	1.14	13	-4.27	$9 \cdot 10^{-4}$

Reported statistics were obtained by applying the same analysis as in the main manuscript, but using a single RT bin. The "*N* effect" shows the number of observers with a positive bias for the prior-dependent experiments, or with a negative bias for the context-dependent experiments.

Supplementary Table 3 – MTurk observer exclusion criteria.

	Discrimination MTurk		TI MTurk until-response		TI MTurk 200 ms		TI MTurk mix	
**Exclusion rule**	**Criterion**	N_{rej}/N_{tot}	**Criterion**	N_{rej}/N_{tot}	**Criterion**	N_{rej}/N_{tot}	**Criterion**	N_{rej}/N_{tot}
Mean RT >	350 ms	0/84	450 ms	0/63	450 ms	2/70	450 ms	14/647
Mean RT in the fastest block >	350 ms	0/84	450 ms	2/63	450 ms	9/70	450 ms	65/647
Mean accuracy >	75%	6/84	65%	1/63	65%	2/70	65%	31/647
Mean accuracy in the worst block >	75%	18/84	65%	2/63	65%	5/70	65%	72/647
Reduction in the mean accuracy from the best to the worst block <	0.2	16/84	0.2	2/63	0.2	3/70	0.2	31/647
Response heterogeneity <	26%	31/84	26%	4/63	26%	11/70	17% for n=4<br>25% for n=8<br>26% for n>8	137/647
Combination of all rules	-	34/84	-	5/63	-	17/70	-	172/647

Shown are the criteria used to exclude MTurk observers, as well as the counts of rejected observers per criterion out of the total observer pool. The exclusion criteria were pre-determined, except for the "mix" dataset. Accuracy refers to the percentage of trials that measured a correct response, excluding the impossible trials (targets oriented 0°), and chance level is 50%. Response heterogeneity is the mean probability that the observer provided a different response for identical stimuli, where the identity requires all the following: the same target orientation, the same context type (in the "mix" experiments having different context types), and the same context orientation(s). Specifically, given a response probability, p, measured for a set of identical stimuli, of size n, the response heterogeneity was $2p(1-p)$, averaged across all sets of identical stimuli. The threshold for heterogeneity depends on the number of repetitions with identical stimuli, n, as seen in the table. The heterogeneity heuristic was typically more sensitive than the performance accuracy for identifying uncooperative observers.

Supplementary Method 1 - TI "mix" dataset

To verify that findings are robust and easily replicable, we analyzed a larger dataset, pooling data from a set of experiments obtained through the Amazon Mechanical Turk. The experiments investigated spatial properties of the TI, and the findings are planned to be reported separately. Here we provide only the RT analysis relevant to the present work.

Stimuli and task

Stimuli consisted of a target sine-wave circle as in the other TI experiments (oriented from -9° to +9° in steps of 1°), and "near-surround" sine-wave annulus similar to the single surround annulus described in the main Methods section, and a "far-surround" sine-wave annulus. The near-surround annulus was full, partial, or empty, and was oriented -90°, -45°, -20°, 0°, +20°, or +45° (trials with an empty, -90° tilted, or 0° tilted near-surround were ignored in analysis). A partial near-surround was either a single half (up or down), a single quadrant (left, right, up, or down), two quadrants (left and right, or up and down), or four quadrants (left, right, up, and down with smoothed surrounding edges). The size of the near-surround was ~1.6%, ~3.3%, ~6.6%, ~13.2%, or ~26.4% of screen height. The gap between the near and the far surrounds was ~1% of screen height. The far-surround annulus was oriented -90°, -20°, 0°, or +20°, had a size of ~16.6% of screen height (if presented), and was always ignored in analysis. Phase was separately randomized for each stimulus component (target, near surround, quadrants of the near surround, and far surround). The near and far sine-wave gratings had an amplitude of 32, 64, or 128 gray levels (corresponding to a contrast of 25%, 50%, or 100%, respectively, in a linearized display). The stimuli were presented either starting from 350 ms after the trial initiation, or starting from 450 ± 100 ms after trial initiation (onset jitter). Presentation duration was 100, 200, 400 ms, or until-response. The task was identical to the task used in the other TI experiments.

Procedure

Observers performed a single session consisting of four or five blocks, each containing between 152 and 228 trials. Data were collected using the Amazon Mechanical Turk from $N = 475$ observers, with additional $N = 172$ MTurk excluded observers following the criteria in Supplementary Table 3. The exclusion criteria were determined post-hoc using a small pool of

observers (therefore, they were not pre-determined). Procedural details were otherwise identical to the TI experiments reported in the main Methods section

Supplementary References

1. Ratcliff, R. & Rouder, J. N. Modeling response times for two-choice decisions. *Psychol. Sci.* **9**, 347–356 (1998).

2. Pinchuk-Yacobi, N., Dekel, R. & Sagi, D. Expectations and visual aftereffects. *J. Vis.* **16**, 19 (2016).
