# Supplementary material for: Perceptual bias is reduced with longer reaction times during visual discrimination
Source: Commun Biol. 2020 Feb 10;3:59. doi: 10.1038/s42003-020-0786-7 (PMC7010686; doi:10.1038/s42003-020-0786-7)
Supplement: Supplementary file 3 — Description of Additional Supplementary Files [file 42003_2020_786_MOESM3_ESM.pdf]

Description of additional supplementary files

Supplementary data 1. Source behavioral data.

See “Figure <N>” tabs for source behavioral data of the main figures.

See additional tabs for individual data (titles following experimental names). Shown, for each observer in four RT bins, are (i) the bias or the average bias (equation (1)), (ii) the average RT in the trials used to calculated the bias, (iii) the *d*’ or the average *d*’ (equation (3)), (iv) the average RT in the trials used to calculate the *d*’, and (v) an approximation of the maximum bias that can be measured (as determined by the number of trials in the relevant conditions of the experiment).
