# Supplementary material for: Perceptual bias is reduced with longer reaction times during visual discrimination
Source: Commun Biol. 2020 Feb 10;3:59. doi: 10.1038/s42003-020-0786-7 (PMC7010686; doi:10.1038/s42003-020-0786-7)
Supplement: Supplementary file 4 — Reporting Summary [file 42003_2020_786_MOESM4_ESM.pdf]

nature research

Corresponding author(s): Dov Sagi

Last updated by author(s): Jan 9, 2020

Reporting Summary

Nature Research wishes to improve the reproducibility of the work that we publish. This form provides structure for consistency and transparency in reporting. For further information on Nature Research policies, see Authors & Referees and the Editorial Policy Checklist.

Statistics

For all statistical analyses, confirm that the following items are present in the figure legend, table legend, main text, or Methods section.

n/a	Confirmed	
☐	☒	The exact sample size (n) for each experimental group/condition, given as a discrete number and unit of measurement
☐	☒	A statement on whether measurements were taken from distinct samples or whether the same sample was measured repeatedly
☐	☒	The statistical test(s) used AND whether they are one- or two-sided<br>*Only common tests should be described solely by name; describe more complex techniques in the Methods section.*
☐	☒	A description of all covariates tested
☐	☒	A description of any assumptions or corrections, such as tests of normality and adjustment for multiple comparisons
☐	☒	A full description of the statistical parameters including central tendency (e.g. means) or other basic estimates (e.g. regression coefficient) AND variation (e.g. standard deviation) or associated estimates of uncertainty (e.g. confidence intervals)
☐	☒	For null hypothesis testing, the test statistic (e.g. F, t, r) with confidence intervals, effect sizes, degrees of freedom and P value noted<br>*Give P values as exact values whenever suitable.*
☒	☐	For Bayesian analysis, information on the choice of priors and Markov chain Monte Carlo settings
☒	☐	For hierarchical and complex designs, identification of the appropriate level for tests and full reporting of outcomes
☐	☒	Estimates of effect sizes (e.g. Cohen's d, Pearson's r), indicating how they were calculated

Our web collection on statistics for biologists contains articles on many of the points above.

Software and code

Policy information about availability of computer code

Data collection	Stimuli presentation and reply collection was achieved using dedicated software. The laboratory prior-dependent experiments (detection, discrimination), and all TAE experiments, were performed with a dedicated C++ software using OpenGL. The TI experiments (laboratory and MTurk), and the prior-dependent discrimination experiment through MTurk, were performed with a dedicated Javascript software, running on a web browser, and using WebGL
Data analysis	Simple statistical analysis (t-tests, linear mixed regression analysis, least-squares fitting), as well as computing the probability density function of the standard drift diffusion model (DDM, Ratcliff, 1978) (equation (6)), was achieved with the MATLAB 2019b software. To compute the probability density function of the DDM that has inter-trial variability parameters (Supplementary Fig. 9), we used the Fast-DM software (Voss & Voss, 2007). Fitting of psychometric function for the purpose of finding the perceived orientation was achieved by using the Psignifit 3.0 software (Fründ, Haenel, & Wichmann, 2011).

For manuscripts utilizing custom algorithms or software that are central to the research but not yet described in published literature, software must be made available to editors/reviewers. We strongly encourage code deposition in a community repository (e.g. GitHub). See the Nature Research guidelines for submitting code & software for further information.

Data

Policy information about availability of data

All manuscripts must include a data availability statement. This statement should provide the following information, where applicable:

- Accession codes, unique identifiers, or web links for publicly available datasets
- A list of figures that have associated raw data
- A description of any restrictions on data availability

The data that support the findings of this study are available from the corresponding author upon reasonable request

Field-specific reporting

Please select the one below that is the best fit for your research. If you are not sure, read the appropriate sections before making your selection.

☐ Life sciences ☒ Behavioural & social sciences ☐ Ecological, evolutionary & environmental sciences

For a reference copy of the document with all sections, see nature.com/documents/nr-reporting-summary-flat.pdf

Behavioural & social sciences study design

All studies must disclose on these points even when the disclosure is negative.

Study description

Quantitative experimental study. The experiments were performed in a laboratory, or with a web-based interface through the Amazon Mechanical Turk (MTurk).

Research sample

Laboratory: N = 43 subjects participated (32 females, 11 males, aged 26 ± 4, Mean ± SD, median of 25, in the range 18-40), including data from eleven subjects obtained with permission from Pinchuk et al. 2016. All participants live at or near the city of Rehovot, Israel. Most participants were students of the Rehovot Campus of the Hebrew University of Jerusalem (i.e., Faculty of Agriculture, Food and Environment). Observers were recruited by advertisement, and began participation in the experiments conditioned on passing an eye examination. Most observers had prior experience in participating in psychophysical experiments.

MTurk: N = 864 subjects participated (of which N = 228 were excluded; reported age of 36 ± 11, median of 33, in the range 18-74; gender information was not always collected; additional observers were excluded, as described below). The typical selection criteria were: 'PercentAssignmentsApproved' ≥ 99, 'NumberHITsApproved' ≥ 2000, 'LocaleCountry' = 'US'.

Sampling strategy

Laboratory: the sample size was based on availability of participants (the sampling procedure was 'convenience'). There were at least seven participants per experiment, which we believe is representative and sufficient for the purpose of studying basic visual phenomena using within-individual effects that are robust in the sampled population. As described in the Methods, we employ psychophysical methods that are standard in vision research. The perceptual effects studied are strong and are expected to be present in most, if not all, tested subjects. In this type of research the subjects are tested many times each, gaining experience with the task, so the results are expected to be stable. The detailed statistical analysis described in the text confirms the robustness of the results.

MTurk: we estimated, using power analysis based on the laboratory study and our experience with the reliability of subjects in the MTurk platform, that 10-15 observers would be sufficient per experiment. We decided on a much larger sample (pre-determined 30-50 subjects per experiment), for the purpose of ensuring that the statistical results are overwhelmingly convincing.

Data collection

Data were collected using a computer, with stimuli being presented on a monitor, and replies being collected using a keyboard. In the laboratory, during response collection, there was no one present beside the participant. The researcher was not blind to the purpose of the experiments, but all observers were naïve to the purpose of the experiments.

Timing

Data collection start date: January 2017, stop date: October 2019.

Data exclusions

Laboratory:
Two observers in the Discrimination experiment were disqualified prior to RT-based data analysis, one having anomalously high accuracy ($d' = 2.5$, with other observers showing $d' = 1.01 \pm 0.18$, mean ± STD), and the other having an anomalously strong baseline response bias in favor of one of the alternatives, which saturates the measured probabilities. Both disqualified observers exhibited a prior-dependent bias in fast replies and no prior-dependent bias in slow replies. Note that the design of this specific experiment assumes that all observers have comparable sensitivity. These criteria were not pre-established.
Also, daily sessions were pruned based a judgment call by the researcher, made prior to data analysis, to address cases where the observer reported a "bad session" and/or the observer was suspect of not complying with the task instructions in some way (TAE Fixation experiment: one observer, Detection experiment: one observer). This criterion was pre-established.
In all experiments, isolated trials were pruned, as described in the Methods section, for the purpose of addressing rare lapses, and for the purpose of ignoring trials performed before the experimental priors could be learned. All claimed findings remained significant in the absence of any trial exclusions. These criteria were not pre-established.

MTurk:
Exclusion criteria were as described in Supplementary Table 3. For most experiments, the criteria were pre-established, the only exception being the "mix" dataset (see the Methods).

Non-participation

Laboratory:
One observer in the TAE periphery experiment dropped out, after her first daily session, and her data were not analyzed. The reason given: I found a new job and will not have time to complete the experiment in the foreseeable future. No participants declined participation.

MTurk:
By the nature of the platform, participants may stop performing the experiment at will, without providing a justification. Of the participants who "Accepted" the "HIT", roughly 37% completed the offered experiment and received compensation.

Randomization

Participants were not allocated into experimental groups. Each participant could take part in multiple experiments of this study. The findings in this study are within-individual effects.

Reporting for specific materials, systems and methods

We require information from authors about some types of materials, experimental systems and methods used in many studies. Here, indicate whether each material, system or method listed is relevant to your study. If you are not sure if a list item applies to your research, read the appropriate section before selecting a response.

Materials & experimental systems

n/a	Involved in the study
☒	☐ Antibodies
☒	☐ Eukaryotic cell lines
☒	☐ Palaeontology
☒	☐ Animals and other organisms
☐	☒ Human research participants
☒	☐ Clinical data

Methods

n/a	Involved in the study
☒	☐ ChIP-seq
☒	☐ Flow cytometry
☒	☐ MRI-based neuroimaging

Human research participants

Policy information about studies involving human research participants

Population characteristics	See above.
Recruitment	Laboratory: Participants were recruited by advertisement, and began participation in the experiments conditioned on passing an eye examination. Participants were compensated at a rate of 50 NIS per daily session.<br>MTurk: participants were recruited through the MTurk platform. The offered compensation was calculated based on an approximate ~$7.5/hour rate.
Ethics oversight	The experiments in this study were approved by the Institutional Review Board (IRB) of the Weizmann Institute of Science, Israel.

Note that full information on the approval of the study protocol must also be provided in the manuscript.
